# The Japan Registry for Adult Subjects of Spinal Muscular Atrophy (jREACT-SMA): Protocol for a Longitudinal Observational Study

**DOI:** 10.2196/38878

**Published:** 2022-12-15

**Authors:** Kentaro Sahashi, Atsushi Hashizume, Yachiyo Kuwatsuka, Madoka Chinen, Ai Saotome-Nakamura, Masahiko Ando, Masahisa Katsuno

**Affiliations:** 1 Department of Neurology Nagoya University Graduate School of Medicine Nagoya Japan; 2 Department of Clinical Research Education Nagoya University Graduate School of Medicine Nagoya Japan; 3 Department of Advanced Medicine Nagoya University Hospital Nagoya Japan; 4 Biogen Japan Ltd Tokyo Japan

**Keywords:** adult, disease-modifying therapy, Japan, nusinersen, observational study, registry, spinal muscular atrophy

## Abstract

**Background:**

Spinal muscular atrophy (SMA) is an autosomal recessive genetic neuromuscular disorder with progressive muscle weakness and atrophy, mainly caused by lower motor neuron degeneration resulting from decreased levels of the survival motor neuron protein. Recently, 3 disease-modifying therapies for SMA (nusinersen, onasemnogene abeparvovec, and risdiplam) were approved in Japan that are expected to improve the prognosis of patients with SMA. Long-term clinical follow-up of adult patients treated with disease-modifying therapies and the natural history of SMA are essential to assess the real-world effectiveness of available treatments. Until recently, nusinersen was the only treatment option for patients with SMA in Japan; however, because Japanese approval of nusinersen was based on global clinical trials in infants and children aged 0-15 years with SMA, the effectiveness of nusinersen in adult patients has not been fully assessed in Japan. In addition, longitudinal clinical data of adult patients have not been systematically collected in Japan.

**Objective:**

This longitudinal observational study of adult patients with SMA who have been diagnosed with 5q-SMA in Japan aims to gain a better understanding of the natural history of SMA, as well as the long-term effectiveness of disease-modifying therapies. Here, we describe the protocol for the study.

**Methods:**

The Japan Registry for Adult Subjects of Spinal Muscular Atrophy (jREACT-SMA) study is a longitudinal (prospective and retrospective) observational study with a 60-month prospective follow-up being conducted at 19 investigational sites using the newly established jREACT-SMA registry. Patients aged ≥18 years with genetically confirmed 5q-SMA were planned to be enrolled in the registry from December 2020 to May 2022. The planned enrollment was 100 patients. The protocol was approved on September 28, 2020 (approval 2020-0289) by the ethical review committee of Nagoya University. Registration, demographics, genetic diagnosis, motor functions, patient-reported outcomes/quality-of-life outcomes, and other clinical data have been or will be collected.

**Results:**

As of May 2022, 113 patients had been enrolled, and the completion of patient registration has been extended from May 2022 to December 2022. Data at registration and during the follow-up period were and will be prospectively collected at least once a year until November 2025 (maximum 60 months). Data analyses will be conducted when all data have been collected. Results are expected to be available in 2026 and the study is expected to be completed by March 2027.

**Conclusions:**

This jREACT-SMA study will provide longitudinal prospective follow-up data in adult patients with SMA in Japan, including data on the natural history of the disease and data on the long-term effectiveness of disease-modifying therapies.

**Trial Registration:**

University Hospital Medical Information Network Center Clinical Trials Registry UMIN000042015; https://rctportal.niph.go.jp/en/detail?trial_id=UMIN000042015

**International Registered Report Identifier (IRRID):**

DERR1-10.2196/38878

## Introduction

Spinal muscular atrophy (SMA) is an autosomal recessive genetic neuromuscular disorder with progressive muscle weakness and atrophy mainly caused by lower motor neuron degeneration resulting from decreased levels of the survival motor neuron (SMN) protein [1,2]. The *SMN* gene is an SMA-determining gene mapped to chromosome 5q13 [3]. Patients with 5q-SMA have the loss of *SMN1* or an *SMN1* point mutation and at least one copy of the highly homologous gene, *SMN2* [3,4]. *SMN2* differs from *SMN1* by only 11 nucleotides (7 in intron 6, 2 in intron 7, 1 in coding exon 7, and 1 in noncoding exon 8) [3,5-7], resulting in the alteration of splicing regulation and exclusion of exon 7 [5]. *SMN2* produces lower levels of the full-length *SMN* transcript [8], whereas *SMN* exon 7–skipped mRNA produces unstable protein, which is rapidly degraded [9].

SMA is one of the rare diseases affecting patients across a broad age range that is designated as an intractable disease [10] and a specific pediatric chronic disease [11]. The incidence of infantile-onset SMA (type I SMA) in Japan is 0.27 per 10,000 live births, and the number of Japanese patients with SMA is assumed to be approximately 1500 [12].

Before 2017, no disease-modifying therapy was available that could influence the clinical course of SMA [13]. Because patients with a higher copy number of *SMN2* tend to develop SMA with later onset and/or show relatively milder symptoms [9,14], increasing the level of full-length functional SMN protein from *SMN2* is expected to alleviate symptoms in patients with SMA [15]. Nusinersen is an antisense oligonucleotide drug that selectively corrects the splicing of *SMN2* pre-mRNA to produce increased amounts of functional SMN protein [15,16]. The efficacy and safety of nusinersen were demonstrated in clinical trials (some including Japanese patients) in infants and children aged 0-15 years with SMA [17-21]. A clinical trial to assess the efficacy of an investigational higher dose of nusinersen in patients with SMA, including patients aged ≥18 years, is also ongoing (ClinicalTrials.gov NCT04089566). In Japan, nusinersen was approved for the treatment of infantile-onset type I SMA in July 2017, and subsequently, its indications were expanded to later-onset types II, III, and IV SMA. In addition, onasemnogene abeparvovec, a gene replacement therapy, was approved in March 2020 for patients with SMA aged under 2 years old, and risdiplam, an oral, once-daily splicing modifier, was approved in June 2021 for patients aged 2 months or older.

The natural history of SMA has changed over the last decade due to improvements in care; in particular, the survival of critically ill infants with type I SMA has increased [22,23]. In addition, these new treatments are expected to improve the prognosis of SMA, suggesting that the number of adult patients with SMA will possibly increase. Neuromuscular symptoms, including muscle weakness, progress more slowly in adult patients with later-onset SMA compared with patients with infantile-onset SMA [24,25]. Therefore, long-term clinical follow-up of treated patients and the natural history of SMA are essential to assess the effectiveness of the treatment, particularly the improvement and/or maintenance of motor functions. Registry and multicenter observational studies of SMA that include adult patients have been reported or are ongoing in several countries [26-30]. At the planning and launch stage of this research, nusinersen was the only treatment option for patients with SMA in Japan. However, the administration regimen of nusinersen for later-onset SMA is different between Japan and other countries. In Japan, the treatment regimens used in the ENDEAR study [18] (4 loading doses and once every 4 months for the maintenance dose with adjustment for age) and in the CHERISH study [19] (3 loading doses and once every 6 months for the maintenance dose) have been approved for infantile-onset SMA and later-onset SMA, respectively. However, in other countries, only the ENDEAR treatment regimen has been approved for all types, including later-onset SMA. As such, the effectiveness of nusinersen in adult patients has not been fully assessed in Japan in both clinical trials and clinical practice. Therefore, it is important to systematically collect longitudinal clinical data, including the natural history of SMA in adult patients. As more treatment options have become available, we expect to gain a better understanding of the pathophysiology of SMA and clinical data to support treatment decisions obtained from long-term clinical follow-up in adult patients with and without treatment.

Here, we describe the protocol for a longitudinal observational study in adult patients with SMA who have been diagnosed with 5q-SMA, which is being conducted with the aim of establishing a new registry (Japan Registry for Adult Subjects of Spinal Muscular Atrophy [jREACT-SMA]). The study will lead to a better understanding of the natural history of SMA, as well as the long-term effectiveness of disease-modifying therapies in Japan.

## Methods

### Study Design

**Figure 1:**
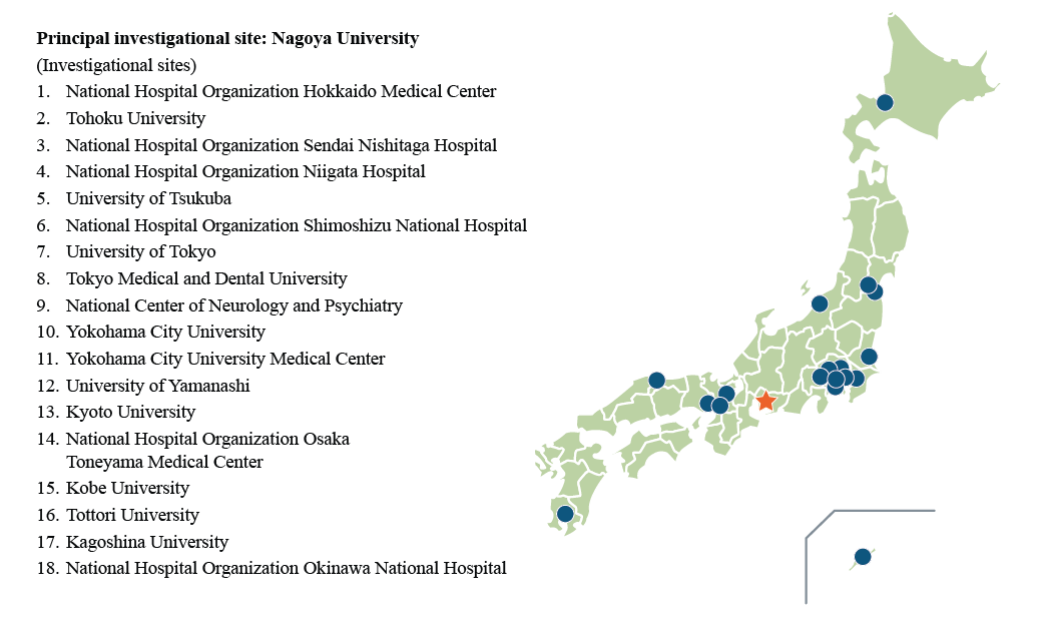
Collaborative investigational sites of the jREACT-SMA study in Japan. The star indicates the principal investigational site (Nagoya University). The other collaborative investigational sites are indicated by circles. jREACT-SMA: Japan Registry for Adult Subjects of Spinal Muscular Atrophy.

The jREACT-SMA study is a longitudinal (prospective and retrospective) observational registry study with a 60-month prospective follow-up being conducted at 19 investigational sites in Japan, including university hospitals, specialist centers, and tertiary hospitals (Figure 1). Patient data are collected from multiple centers because treatment for SMA is not centralized in Japan. The planned number of patients was 100, which takes into consideration the feasibility of enrolling patients; however, registration of additional patients is permitted (Textbox 1).

**Study plan.**

**Study design**

- Multicenter, prospective and retrospective, and observational study

**Study population**

- Patients aged ≥18 years, with diagnosed spinal muscular atrophy (SMA) and genetically confirmed deletion or mutations of the *survival motor neuron* (*SMN1*) gene (5q-SMA) as well as at least 1 copy of the *SMN2* gene

**Planned number of patients**

- 100

**Data to be collected**

- Motor functions
- Measurement of motor abilities
- Physiological test (eg, respiratory function test)
- Blood test
- Patient-reported outcome and quality of life

**Registration period (patient registration was planned to end in May 2022 and has been extended to December 2022)**

- From December 2020 to May 2022 (18 months)

**Prospective follow-up period**

- From December 2020 to November 2025 (60 months)

### Sample Selection

Patients who fulfill the following inclusion criteria are eligible for enrollment (Textbox 1): diagnosed with SMA, as defined by the Diagnostic Criteria of the Research Committee for Spinal Muscular Atrophy from the Ministry of Health, Labour and Welfare of Japan [31]; aged ≥18 years; genetically confirmed deletion or mutations of the *SMN1* gene (5q-SMA), as well as at least 1 copy of the *SMN2* gene; able to attend the investigational sites at least once a year during the follow-up period; and able to understand the purpose of the study and provide written informed consent. Patients who had any condition (such as psychiatric disorders) that would make it difficult to comply with study requirements and patients who were deemed by the investigators to be unsuitable for study enrollment are excluded.

Patients will be enrolled regardless of treatment status. The requirement for treatment is decided by the investigators independently from the study, taking into account each patient’s preference and medical condition. Disease-modifying therapies are administered according to the approved administration regimen.

### Measurements and Planned Outcomes

Motor function scales and patient-reported outcomes (PRO)/quality-of-life (QOL) outcomes are the primary end points of the study. By partly referring to the TREAT-NMD SMA Registries Core Dataset [32], registration, demographics, genetic diagnosis, motor functions, PRO/QOL data, and other clinical data have been or will be collected (Table 1, data that are highly encouraged to be collected are shown in italics). All data have been or will be obtained from medical records or at regular visits. Further details and the schedule of data collection are shown in Table 1, and free-text columns are available for investigators to collect data other than those listed. Any adverse events for patients treated with each of the disease-modifying therapies are to be reported according to the standard safety report procedures in the respective all-case surveillance studies.

**Table 1:** Schedule of data collection. Highly encouraged data to be collected are expressed in italics.

Data to be collected (categories)	Variables	Retrospective observation period (from first visit)	Baseline (at registration; day −30 to registration)	Follow-up period (prospective observation period; registration to 60 months)
Informed consent	*Date of registration* *Date of informed consent* *Registration number*		✓	
Inclusion/exclusion criteria	N/A^a^		✓	
Demographics	*Age at registration* or date of birth *Sex*		✓	
Survival status	*Survival status (age at death and causes of death if applicable)*			✓
Genetic diagnosis^b^	*Name of genetic testing center* *Genetic test results* (SMN1^c^ *exon 7/exon 8 copy number*, SMN1 *deletion/mutation, and* SMN2 *exon 7/exon 8 copy number)*	✓	✓	
Clinical findings	*Age at symptom onset* *SMA^d^ type (I, II, III, or IV)* *Body weight* Height	✓^e^	✓	✓^e^
Scoliosis	*Presence or absence (surgical history, age, and surgical procedure at the first surgery if applicable)*		✓	
Motor functions (“able to do” or “unable to do” for each function)	*Maintaining head upright without support* *Rolling to side* *Sitting without support* *Crawling on hands and knees* *Standing with support* *Standing without support* *Walking independently* *Walking 10 m independently* *Going up the stairs* *Using whole hands* *Raising hands overhead in a sitting position* *Raising hands to mouth in a sitting position*	✓	✓	✓
Wheelchair use	*Wheelchair use (age started if applicable)*	✓	✓	✓
Nutrition	*Tube feeding (age started if current; age ended if previous)*	✓	✓	✓
Artificial ventilation^f^	*Invasive/noninvasive ventilation (age started, hours/day if applicable)*	✓	✓	✓
Medications	*Medications for SMA* (if applicable, collect the information below) Name of drugs (nusinersen sodium, onasemnogene abeparvovec, risdiplam, sodium valproate, or others) Age started medication Date started medication Dosage per administration and administration date if nusinersen Age at discontinuation and reasons for discontinuation	✓	✓	✓
Hospitalizations (except medication purpose) and comorbidities	*Hospitalization in last 12 months from registration and between the previous visit and the latest visit during follow-up period (date of hospitalizations and name of disease if applicable)* *Comorbidities diagnosed in last 12 months from registration and between the previous visit and the latest visit during follow-up period, other than diseases that are reasons for hospitalization (age at onset of comorbidities or age fully recovered if applicable)*	✓^g^	✓	✓
Clinical trials	*Clinical trials for SMA (name of investigational drugs and age participated if applicable)*	✓	✓	✓
Measurement of motor abilities	*2MWT^h^ (m)^i^* *6MWT^j ^(m)^i^* *HFMSE^k^ (total score)* *RULM^l^ (higher score of left/right)* Pinch strength (kg) Grip strength (kg) Tongue pressure (kPa) MRC^m^ score (higher score of left/right, grade 0, 1, 2, 3, 4, 5) Iliopsoas muscle Deltoid muscle Biceps brachii muscle Triceps brachii muscle Quadriceps femoris muscle Cervical flexor muscle Hamstring muscle Tibialis anterior muscle Gastrocnemius muscle	✓	✓	✓
X-ray examination	Quantitative bone mineral test Skeletal muscle mass by DXA^n^ (kg)	✓	✓	✓
Physiological test	Nerve conduction test Ulnar nerve CMAP^o^ (mV or uV) Respiratory function test *FVC^p^* *VC^q^* *% VC* *FEV1.0^r^* *FEV1.0%^s^* Peak cough flow	✓	✓	✓
Blood test	*Creatine kinase* *Creatinine* *Creatine* *Cystatin C*	✓	✓	✓

PRO^t^/QOL^u^	*mSMAFRS^v^ (total score)* ALSFRS-R^w^ (total score) MFI-20^x^ (total score) SDQ^y^ (total score) *CGI-S^z^* *CGI-I^aa^* *TGI^ab^* *mRS^ac^* Other validated PROs	✓	✓	✓
Rehabilitation	*Rehabilitation (age started if current or age ended if previous)*	✓	✓	✓

^a^N/A: not applicable.

^b^Confirmed at registration.

^c^SMN: survival motor neuron.

^d^SMA: spinal muscular atrophy.

^e^Weight only.

^f^Collected data over time.

^g^Collected data for the last 12 months from registration.

^h^2MWT: 2-minute walk test.

^i^Highly encouraged to assess either one for ambulant patients.

^j^6MWT: 6-minute walk test.

^k^HFMSE: Hammersmith Functional Motor Scale–Expanded.

^l^RULM: Revised Upper Limb Module.

^m^MRC: Medical Research Council.

^n^DXA: dual-energy X-ray absorptiometry.

^o^CMAP: compound muscle action potential.

^p^FVC: forced vital capacity.

^q^VC: vital capacity.

^r^FEV1.0: forced expiratory volume in 1 second.

^s^FEV1.0%: forced expiratory volume % in 1 second.

^t^PRO: patient-reported outcome.

^u^QOL: quality of life.

^v^mSMAFRS: Modified SMA Functional Rating Scale.

^w^ALSFRS-R: Amyotrophic Lateral Sclerosis Functional Rating Scale-Revised.

^x^MFI-20: Multidimensional Fatigue Inventory-20.

^y^SDQ: Swallowing Disturbance Questionnaire.

^z^CGI-S: Clinical Global Impressions–Severity scale.

^aa^CGI-I: Clinical Global Impressions–Improvement scale.

^ab^TGI: Total Global Impression.

^ac^mRS: modified Rankin Scale.

### Data Collection

The jREACT-SMA study uses the Research Electronic Data Capture system, operated by the ARO Data Coordinating Center, Department of Advanced Medicine, Nagoya University Hospital (Nagoya, Japan) and the Department of Neurology, Nagoya University Graduate School of Medicine (Nagoya, Japan), to register patients and collect/centralize data. The Research Electronic Data Capture system can be accessed securely by the investigators and study-related personnel only.

Information that can identify patients is anonymized at data entry. Anonymized data are labeled with an identifying code to reidentify patients. The identifying codes linking patients with their anonymized data are securely stored at each investigational site. The investigators are responsible for saving source data and ensuring the quality of data at each investigational site.

### Data Analysis

Demographics at baseline and clinical characteristics at first visit will be summarized. Time-to-event outcomes (survival status and time to tracheostomy) will be analyzed using the Kaplan-Meier method. Continuous variables will be summarized by descriptive statistics, including arithmetic mean, standard deviation, minimum, 25% quartile, median, 75% quartile, maximum, and proportion of missing values. Categorical variables will be analyzed using Wilcoxon signed-rank test with the number and percentage in each category. Additional analyses referring to specific research questions may be conducted and will be described in a statistical analysis plan. The statistical analysis plan will be completed by database lock. Interim and final analyses are planned.

### Ethics and Dissemination

The study is being conducted in compliance with the Declaration of Helsinki, Ethical Guidelines for Medical and Health Research Involving Human Subjects, and the Act on the Protection of Personal Information. Written informed consent has been or will be obtained from all patients, including agreement for publication. If patients withdraw informed consent, the patients’ data will be excluded from the data set as much as possible. The study is registered at the University Hospital Medical Information Network Center Clinical Trials Registry (UMIN000042015). Collaborative investigational sites must obtain approval from their own relevant ethics committees before starting the study. Prior to patient screening, a contract research organization (Mebix, Inc.), funded by Biogen Japan Ltd, reviewed study responsibilities with the investigators and study-related personnel.

### Ethics Approval

The study is led by Nagoya University, and the protocol was approved on September 28, 2020 (approval 2020-0289) by the ethical review committee of Nagoya University.

## Results

**Figure 2:**
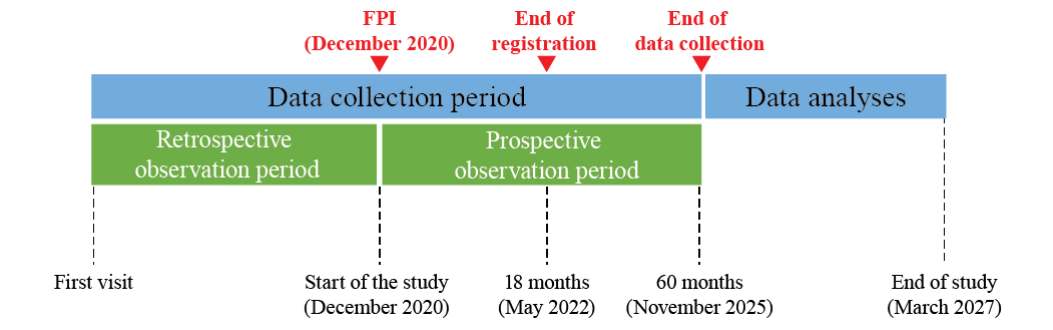
Study design. The study started and the first patient was registered in December 2020. Patient registration was planned to end in May 2022 and has been extended to December 2022. Retrospective data from their first visit to registration will be collected from medical records, and prospective data will be collected at regular visits until November 2025. Data analyses are planned after data collection is complete, and the study is expected to end in March 2027. FPI: first patient in (date of the first patient registration).

Patient registration started in December 2020 and was planned to end in May 2022 (Textbox 1 and Figure 2). As of May 2022, 113 patients had been enrolled, and patient registration has been extended to December 2022. Data from the first visit (first visit at the investigational site regardless of diagnosis, with or without treatment; data before diagnosis could be inputted) to registration were and will be retrospectively collected. Data at registration and during the follow-up period were and will be prospectively collected at least once a year until November 2025 (maximum 60 months). Data analyses will be conducted when all data have been collected. Results are expected to be available in 2026 and the study is expected to be completed by March 2027 (Figure 2).

## Discussion

### Expected Findings

This protocol is for the first longitudinal (prospective and retrospective) observational study in Japanese adult patients with SMA, regardless of treatment status, with the aim of establishing a new registry named jREACT-SMA. Using the results from the jREACT-SMA study, we expect to gain a better understanding of the pathophysiology of SMA and clinical data to support treatment decisions for overcoming potential therapeutic limitations in adult patients with SMA.

Outside of Japan, other registry or observational studies have been or will be reporting real-world clinical data—including motor function scales such as the 6-minute walk test, the Hammersmith Functional Motor Scale–Expanded, and the Revised Upper Limb Module—in adult patients with SMA [26-30]. In addition, some of these studies will provide PRO and sociodemographic data [28]. However, data in Japanese patients with SMA are still required because the administration regimen of nusinersen for later-onset SMA is different between Japan and other countries. Several registries of patients with SMA are currently in operation in Japan, but no data have been reported yet. The Spinal Muscular Atrophy Research & Treatment Consortium [33] has been established for patients with SMA and health care professionals to share information regarding new clinical trials and investigator-initiated trials promptly and to help conduct the trials efficiently. The Rare Disease Data Registry of Japan [34] has been established to centralize clinical information and biological samples from the Japan Agency for Medical Research and Development [35] and the intractable disease research groups of the Ministry of Health, Labour and Welfare. Distinct from these registries in Japan, the jREACT-SMA study will provide longitudinal prospective and retrospective follow-up data in adult patients with SMA, including both adult patients who transitioned from pediatric SMA and patients with adult-onset SMA.

In this jREACT-SMA study, patients who do not wish to receive active treatments are registered, as well as patients who have been or are being treated with disease-modifying therapies. Longitudinal observation of SMA in Japanese adult patients is limited [36], and the inclusion of untreated patients in jREACT-SMA will provide valuable information about the natural history of adults with SMA. In addition, because nusinersen was approved in Japan based on the results of clinical trials in infants and children with SMA who were aged ≤9 years at screening [17-19], the effectiveness of nusinersen in adult patients has not been fully assessed in Japan. As mentioned above, the administration regimen of nusinersen in Japan differs from other countries. The number of loading doses and the administration interval were defined by a pharmacokinetics simulation using data from patients with types II and III SMA. Therefore, information on patients treated with the Japan-approved administration regimen may provide further important insights into the optimization and validation of therapeutic protocols for Japanese adult patients with SMA. The comprehensive analyses from the jREACT-SMA study will allow us to address current gaps in our knowledge of the natural history of SMA, as well as the long-term effectiveness of disease-modifying therapies in adult patients.

### Strengths and Limitations

The jREACT-SMA study is the first multicenter, long-term, longitudinal observational study to establish a registry for Japanese adult patients, which will better reflect real-world clinical settings. Most core hospitals that provide specialized treatment for SMA in Japan were included in the study. The only patients who will be excluded are those who have any condition that would make it difficult to comply with the study requirements and those who are deemed by the investigators to be unsuitable for study enrollment. A broad range of data will be collected, including patient background, clinical characteristics, and clinical measures such as motor functions and PRO/QOL, as well as physiological and blood tests. Only adult patients are eligible for the study; this allows examination of the clinical course of the disease in this population of patients with SMA, whose numbers are expected to increase as pediatric patients survive longer. In addition, both patients treated with disease-modifying therapies and untreated patients have been enrolled, which will enable a better understanding of the natural history of SMA and the long-term effectiveness of the therapies during adulthood.

However, several limitations of the study need to be considered. First, we are not able to include all Japanese patients with SMA. Of note, the number of untreated patients to be registered may not be proportionate to their population in Japan because they make fewer hospital visits and are therefore less likely to be enrolled. Second, we cannot identify patients who have not yet been diagnosed with SMA, which makes it difficult to understand the full context of SMA in Japan from the jREACT-SMA study. Third, we may not be able to obtain information longitudinally if patients change hospitals during the data collection period (eg, if a patient moves from a participating hospital to a nonparticipating hospital, we are not able to obtain complete prospective data). Fourth, the baseline of the outcome measures may not be assessed appropriately or could be partly missing in the case of participants who started disease-modifying therapies before enrollment, meaning that data were collected retrospectively. Additionally, follow-up data could be partly missing in the case of participants who stopped regular visits. Fifth, the planned number of patients, especially untreated patients, is small; therefore, it may be difficult to analyze the effectiveness of disease-modifying therapies compared with the natural history of SMA. Sixth, although PRO/QOL are some of the primary end points, it is difficult to demonstrate that benefits in PRO/QOL result from intervention because of the absence of an age- and SMA type–matched control group in the study and the potential for response bias. Seventh, respiratory management (requirement for invasive/noninvasive ventilatory support) is proposed by physicians based on their treatment policy, knowledge, and experience, and the final decision is made by patients/caregivers. Therefore, outcomes relating to artificial ventilation should be carefully interpreted. Finally, the quantity of data (ie, number of patients with data in each category) may vary for different variables because although data collection is highly encouraged or optional, it is not mandatory.

### Conclusions

The jREACT-SMA study will contribute to a better understanding of the natural history of the disease and the long-term effectiveness of disease-modifying therapies with the aim of establishing a database of Japanese adult patients with SMA.

## Abbreviations

jREACT-SMA: Japan Registry for Adult Subjects of Spinal Muscular Atrophy
PRO: patient-reported outcome
QOL: quality of life
SMA: spinal muscular atrophy
SMN: survival motor neuron

## Data Availability

Data sharing is not applicable to this article as no data sets were generated or analyzed during this study.

## References

[1] Farrar MA, Kiernan MC (2015). The genetics of spinal muscular atrophy: progress and challenges. Neurotherapeutics.

[2] Markowitz JA, Singh P, Darras BT (2012). Spinal muscular atrophy: a clinical and research update. Pediatr Neurol.

[3] Lefebvre S, Bürglen L, Reboullet S, Clermont O, Burlet P, Viollet L, Benichou B, Cruaud C, Millasseau P, Zeviani M (1995). Identification and characterization of a spinal muscular atrophy-determining gene. Cell.

[4] Monani UR, Sendtner M, Coovert DD, Parsons DW, Andreassi C, Le TT, Jablonka S, Schrank B, Rossoll W, Prior T W, Morris G E, Burghes A H (2000). The human centromeric survival motor neuron gene (SMN2) rescues embryonic lethality in Smn(-/-) mice and results in a mouse with spinal muscular atrophy. Hum Mol Genet.

[5] Monani UR, Lorson CL, Parsons DW, Prior TW, Androphy EJ, Burghes AH, McPherson JD (1999). A single nucleotide difference that alters splicing patterns distinguishes the SMA gene SMN1 from the copy gene SMN2. Hum Mol Genet.

[6] Bussaglia E, Clermont O, Tizzano E, Lefebvre S, Bürglen L, Cruaud C, Urtizberea JA, Colomer J, Munnich A, Baiget M (1995). A frame-shift deletion in the survival motor neuron gene in Spanish spinal muscular atrophy patients. Nat Genet.

[7] Hahnen E, Schönling J, Rudnik-Schöneborn S, Zerres K, Wirth B (1996). Hybrid survival motor neuron genes in patients with autosomal recessive spinal muscular atrophy: new insights into molecular mechanisms responsible for the disease. Am J Hum Genet.

[8] Lorson CL, Hahnen E, Androphy EJ, Wirth B (1999). A single nucleotide in the SMN gene regulates splicing and is responsible for spinal muscular atrophy. Proc Natl Acad Sci U S A.

[9] Cho S, Dreyfuss G (2010). A degron created by SMN2 exon 7 skipping is a principal contributor to spinal muscular atrophy severity. Genes Dev.

[10] 脊髄性筋萎縮症（指定難病３). Spinal muscular atrophy (intractable disease 3 designated in Japan). Article in Japanese. Japanese Intractable Diseases Information Center.

[11] 42 脊髄性筋萎縮症. 42: Spinal muscular atrophy. Article in Japanese. Information Center for Specific Pediatric Chronic Diseases, Japan.

[12] Ito M, Yamauchi A, Urano M, Kato T, Matsuo M, Nakashima K, Saito K (2022). Epidemiological investigation of spinal muscular atrophy in Japan. Brain Dev.

[13] Finkel RS, Mercuri E, Meyer OH, Simonds AK, Schroth MK, Graham RJ, Kirschner J, Iannaccone ST, Crawford TO, Woods S, Muntoni F, Wirth B, Montes J, Main M, Mazzone ES, Vitale M, Snyder B, Quijano-Roy S, Bertini E, Davis RH, Qian Y, Sejersen T, Care group SMA (2018). Diagnosis and management of spinal muscular atrophy: part 2: pulmonary and acute care; medications, supplements and immunizations; other organ systems; and ethics. Neuromuscul Disord.

[14] Wirth B, Brichta L, Schrank B, Lochmüller H, Blick S, Baasner A, Heller R (2006). Mildly affected patients with spinal muscular atrophy are partially protected by an increased SMN2 copy number. Hum Genet.

[15] Hua Y, Sahashi K, Hung G, Rigo F, Passini MA, Bennett CF, Krainer AR (2010). Antisense correction of SMN2 splicing in the CNS rescues necrosis in a type III SMA mouse model. Genes Dev.

[16] Hua Y, Vickers TA, Okunola HL, Bennett CF, Krainer AR (2008). Antisense masking of an hnRNP A1/A2 intronic splicing silencer corrects SMN2 splicing in transgenic mice. Am J Hum Genet.

[17] Finkel RS, Chiriboga CA, Vajsar J, Day JW, Montes J, De Vivo DC, Yamashita M, Rigo F, Hung G, Schneider E, Norris DA, Xia S, Bennett CF, Bishop KM (2016). Treatment of infantile-onset spinal muscular atrophy with nusinersen: a phase 2, open-label, dose-escalation study. Lancet.

[18] Finkel RS, Mercuri E, Darras BT, Connolly AM, Kuntz NL, Kirschner J, Chiriboga CA, Saito K, Servais L, Tizzano E, Topaloglu H, Tulinius M, Montes J, Glanzman AM, Bishop K, Zhong ZJ, Gheuens S, Bennett CF, Schneider E, Farwell W, De Vivo DC, ENDEAR Study Group (2017). Nusinersen versus sham control in infantile-onset spinal muscular atrophy. N Engl J Med.

[19] Mercuri E, Darras BT, Chiriboga CA, Day JW, Campbell C, Connolly AM, Iannaccone ST, Kirschner J, Kuntz NL, Saito K, Shieh PB, Tulinius M, Mazzone ES, Montes J, Bishop KM, Yang Q, Foster R, Gheuens S, Bennett CF, Farwell W, Schneider E, De Vivo DC, Finkel RS, CHERISH Study Group (2018). Nusinersen versus sham control in later-onset spinal muscular atrophy. N Engl J Med.

[20] De Vivo DC, Bertini E, Swoboda KJ, Hwu W, Crawford TO, Finkel RS, Kirschner J, Kuntz NL, Parsons JA, Ryan MM, Butterfield RJ, Topaloglu H, Ben-Omran T, Sansone VA, Jong Y, Shu F, Staropoli JF, Kerr D, Sandrock AW, Stebbins C, Petrillo M, Braley G, Johnson K, Foster R, Gheuens S, Bhan I, Reyna SP, Fradette S, Farwell W, NURTURE Study Group (2019). Nusinersen initiated in infants during the presymptomatic stage of spinal muscular atrophy: interim efficacy and safety results from the Phase 2 NURTURE study. Neuromuscul Disord.

[21] Darras BT, Chiriboga CA, Iannaccone ST, Swoboda KJ, Montes J, Mignon L, Xia S, Bennett CF, Bishop KM, Shefner JM, Green AM, Sun P, Bhan I, Gheuens S, Schneider E, Farwell W, De Vivo DC, ISIS-396443-CS2/ISIS-396443-CS12 Study Groups (2019). Nusinersen in later-onset spinal muscular atrophy: long-term results from the phase 1/2 studies. Neurology.

[22] Oskoui M, Levy G, Garland CJ, Gray JM, O'Hagen J, De Vivo DC, Kaufmann P (2007). The changing natural history of spinal muscular atrophy type 1. Neurology.

[23] Wijngaarde CA, Stam M, Otto LAM, van Eijk RPA, Cuppen I, Veldhoen ES, van den Berg LH, Wadman RI, van der Pol WL (2020). Population-based analysis of survival in spinal muscular atrophy. Neurology.

[24] Serra-Juhe C, Tizzano EF (2019). Perspectives in genetic counseling for spinal muscular atrophy in the new therapeutic era: early pre-symptomatic intervention and test in minors. Eur J Hum Genet.

[25] Wijngaarde CA, Stam M, Otto LAM, Bartels B, Asselman F, van Eijk RPA, van den Berg LH, Goedee HS, Wadman RI, van der Pol WL (2020). Muscle strength and motor function in adolescents and adults with spinal muscular atrophy. Neurology.

[26] Lusakowska A, Jedrzejowska M, Kaminska A, Janiszewska K, Grochowski P, Zimowski J, Sierdzinski J, Kostera-Pruszczyk A (2021). Observation of the natural course of type 3 spinal muscular atrophy: data from the polish registry of spinal muscular atrophy. Orphanet J Rare Dis.

[27] Mercuri E, Finkel R, Scoto M, Hall S, Eaton S, Rashid A, Balashkina J, Coratti G, Pera MC, Samsuddin S, Civitello M, Muntoni F, iSMAC Group (2019). Development of an academic disease registry for spinal muscular atrophy. Neuromuscul Disord.

[28] Hodgkinson VL, Oskoui M, Lounsberry J, M'Dahoma S, Butler E, Campbell C, MacKenzie A, McMillan HJ, Simard L, Vajsar J, Brais B, Chapman KM, Chrestian N, Crone M, Dobrowolski P, Dojeiji S, Dowling JJ, Dupré Nicolas, Genge A, Gonorazky H, Hasal S, Izenberg A, Johnston W, Leung E, Lochmüller Hanns, Mah JK, Marerro A, Massie R, McAdam L, McCormick A, Melanson M, Mezei MM, Nguyen CE, O'Connell C, O'Ferrall EK, Pfeffer G, Phan C, Plamondon S, Poulin C, Rodrigue X, Schellenberg KL, Selby K, Sheriko J, Shoesmith C, Smith G, Taillon M, Taylor S, Warman Chardon J, Worley S, Korngut L (2020). A national spinal muscular atrophy registry for real-world evidence. Can J Neurol Sci.

[29] Hagenacker T, Wurster CD, Günther René, Schreiber-Katz O, Osmanovic A, Petri S, Weiler M, Ziegler A, Kuttler J, Koch JC, Schneider I, Wunderlich G, Schloss N, Lehmann HC, Cordts I, Deschauer M, Lingor P, Kamm C, Stolte B, Pietruck L, Totzeck A, Kizina K, Mönninghoff Christoph, von Velsen O, Ose C, Reichmann H, Forsting M, Pechmann A, Kirschner J, Ludolph AC, Hermann A, Kleinschnitz C (2020). Nusinersen in adults with 5q spinal muscular atrophy: a non-interventional, multicentre, observational cohort study. Lancet Neurol.

[30] Maggi L, Bello L, Bonanno S, Govoni A, Caponnetto C, Passamano L, Grandis M, Trojsi F, Cerri F, Ferraro M, Bozzoni V, Caumo L, Piras R, Tanel R, Saccani E, Meneri M, Vacchiano V, Ricci G, Soraru' G, D'Errico E, Tramacere I, Bortolani S, Pavesi G, Zanin R, Silvestrini M, Politano L, Schenone A, Previtali SC, Berardinelli A, Turri M, Verriello L, Coccia M, Mantegazza R, Liguori R, Filosto M, Marrosu G, Siciliano G, Simone IL, Mongini T, Comi G, Pegoraro E (2020). Nusinersen safety and effects on motor function in adult spinal muscular atrophy type 2 and 3. J Neurol Neurosurg Psychiatry.

[31] 3 脊髄性筋萎縮症. 3: Spinal muscular atrophy. Article in Japanese. The Research Committee for Spinal Muscular Atrophy from the Ministry of Health, Labour and Welfare of Japan.

[32] (2018). TREAT-NMD SMA Registries Core Dataset version 1. TREAT-NMD.

[33] Spinal Muscular Atrophy Research & Treatment Consortium.

[34] Rare Disease Data Registry of Japan.

[35] Japan Agency for Medical Research and Development.

[36] Kaneko K, Arakawa R, Urano M, Aoki R, Saito K (2017). Relationships between long-term observations of motor milestones and genotype analysis results in childhood-onset Japanese spinal muscular atrophy patients. Brain Dev.

